# Concomitant heterochromatinisation and down-regulation of gene expression unveils epigenetic silencing of *RELB *in an aggressive subset of chronic lymphocytic leukemia in males

**DOI:** 10.1186/1755-8794-3-53

**Published:** 2010-11-10

**Authors:** Jean-Brice Marteau, Odile Rigaud, Thibaut Brugat, Nathalie Gault, Laurent Vallat, Mogens Kruhoffer, Torben F Orntoft, Florence Nguyen-Khac, Sylvie Chevillard, Hélène Merle-Beral, Jozo Delic

**Affiliations:** 1 Commissariat à l'Energie Atomique et aux Energies Alternatives (CEA) Direction des Sciences du Vivant (DSV) Institut de Radiobiologie Cellulaire et Moléculaire (IRCM), Laboratoire d’Onco-Hématologie (LOH), France; 2 GH Pitié Salpêtrière, Service d’Hématologie Biologique, AP-HP, Université Paris 6, Inserm U543, 47, Bd de l’Hôpital, Paris (75013), France; 3 University of Aarhus, Department of Clinical Biochemistry, Brendstrupgaardsvej 100, Aarhus DK-8200, Denmark; 4 Commissariat à l'Energie Atomique et aux Energies Alternatives (CEA) Direction des Sciences du Vivant (DSV) Institut de Radiobiologie Cellulaire et Moléculaire (IRCM), Laboratoire de Cancérologie Expérimentale (LCE), 18, Av du Panorama, Fontenay-aux-Roses (92265), France; 5 Université René Descartes-Paris V, 4, Av de l'Observatoire, Paris (75006), France; 6 Current Address: Jean Langhorne's Lab, Division of Parasitology, National Institute for Medical Research, The Ridgeway London, NW71AA, UK

## Abstract

**Background:**

The sensitivity of chronic lymphocytic leukemia (CLL) cells to current treatments, both *in vitro *and *in vivo*, relies on their ability to activate apoptotic death. CLL cells resistant to DNA damage-induced apoptosis display deregulation of a specific set of genes.

**Methods:**

Microarray hybridization (Human GeneChip, Affymetrix), immunofluorescent *in situ *labeling coupled with video-microscopy recording/analyses, chromatin-immunoprecipitation (ChIP), polymerase chain reactions (PCR), real-time quantitative PCR (RT-QPCR) and bisulfite genome sequencing were the main methods applied. Statistical analyses were performed by applying GCRMA and SAM analysis (microarray data) and Student's t-test or Mann & Whitney's U-test.

**Results:**

Herein we show that, remarkably, in a resistant male CLL cells the vast majority of genes were down-regulated compared with sensitive cells, whereas this was not the case in cells derived from females. This gene down-regulation was found to be associated with an overall gain of heterochromatin as evidenced by immunofluorescent labeling of heterochromatin protein 1α (HP-1), trimethylated histone 3 lysine 9 (3metH3K9), and 5-methylcytidine (5metC). Notably, 17 genes were found to be commonly deregulated in resistant male and female cell samples. Among these, *RELB *was identified as a discriminatory candidate gene repressed in the male and upregulated in the female resistant cells.

**Conclusion:**

The molecular defects in the silencing of *RELB *involve an increase in H3K9- but not CpG-island methylation in the promoter regions. Increase in acetyl-H3 in resistant female but not male CLL samples as well as a decrease of total cellular level of RelB after an inhibition of histone deacetylase (HDAC) by trichostatin A (TSA), further emphasize the role of epigenetic modifications which could discriminate two CLL subsets. Together, these results highlighted the epigenetic *RELB *silencing as a new marker of the progressive disease in males.

## Background

The CLL is currently incurable and is associated with a high incidence of morbidity and mortality in the elderly. Men are more frequently affected than women (0.6/0.4), develop the disease at a younger age [1,2] and often exhibit a more aggressive form of this disease [3]. Consistent with these observations, CLL cells in men more commonly display no mutations in genes of the immunoglobulin variable heavy chain region (IgV_H_), which is a known indicator of a poor prognosis [4]. When gene expression profiles were previously categorized according to the status of IgV_H _genes, males segregated into a distinct subgroup [5]. The current front line therapies for CLL include fludarabine (nucleoside analogue) or chlorambucil (alkylating agent) both of which should induce apoptosis through DNA damage. Fludarabine treatment *in vivo *induces a gene expression response similar to that induced by the *in vitro *exposure of cells to ionizing irradiation [6] suggesting the common mechanisms achievable by these two treatments.

We previously identified the altered expression of a specific subset of genes in leukemic cells that displayed resistance to DNA damage-induced apoptosis, and defined a clinically distinct, aggressive form of CLL [7]. Other groups have identified genes associated with certain CLL subtypes defined by patient survival and disease staging [8], IgV_H _mutation status [9,10] or CD38-expression [11]. CLL has also been associated with global DNA hypomethylation and a hypermethylation of GC-rich promoter regions [12,13], two aberrant epigenetic events that cause chromatin structural changes and subsequent de-regulated gene expressions [14,15]. DNA methylation of CpG islands in the promoter regions of specific cancer-relevant genes, which often occur concomitantly with covalent modifications of histones and/or with the appearance of their variants, establishes a direct epigenetic basis for cell transformation. Thus, cancer cells display genetic lesions (mutations, deletions and translocations) and significant epigenetic changes that convey heritable gene expression profiles critical for tumorigenesis [16]. In this regard epigenetic control of gene expression has been shown in both sporadic and familial CLL [17].

Based upon the sex-related differences in the occurrence of CLL, the clinical outcomes of this, and the ability to unambiguously distinguish progressive from indolent cases by evaluating the susceptibility to apoptosis after DNA damage *in vitro*, the aim of our present study was to screen for new genes that could discriminate between CLL types classified using these parameters. We used oligonucleotide microarrays to analyze resistant and sensitive CLLs from patients and healthy donors and further validated these results by RT-QPCR. Intriguingly, when compared with sensitive samples, male resistant samples revealed a generalized down-regulation (98%) of gene expression not seen in the corresponding female samples. This characteristic of resistant male CLLs was also associated with a more compact chromatin and more widespread heterochromatic features than in female samples. Furthermore, male and female CLL cell samples shared 17 genes which could distinguish between resistant and sensitive cases. Among these genes, *RELB *was found to be down-regulated in resistant male but up-regulated in female CLL samples. We have now established that the reduced expression of *RELB *in male samples is the result of epigenetic silencing through increased levels of 3metH3K9 in three promoter regions of this gene: region of 58 bp, 121 bp and 74 bp (333-391 bp, 529-650 bp and 1117-1191 bp from transcription initiation site respectively). In parallel, up-regulation of *RELB *in resistant female CLL samples was documented by an increase of acetyl-H3, hallmark of an activated gene expression. Taken together, these results strongly suggest that *RELB *silencing may be involved in the development of resistant subtypes of CLL in males.

## Methods

### Patients and clinical characteristics

Twenty-five CLL samples were selected from our cohort according to their sensitivity to apoptosis. Blood from leukemic (CLL) and healthy donors was collected in heparin-coated tubes or blood-packs after informed consent. B lymphocytes were isolated as previously described [7] with a purity of 86 ± 3% and 95 ± 3% for normal and leukemic B-cells, respectively. The mean percentage of apoptotic cells was determined 24 hours after exposure to 10 Gy (^137^Cs source). For sensitive samples, the level of apoptosis increased from 8.7 ± 1.5% (spontaneous apoptosis) to 73.3 ± 26.3% after irradiation, whereas for resistant samples the apoptosis level of irradiated cells remained unchanged (6.3 ± 3.1% compared to 7.3 ± 3.5% spontaneous apoptotic cells). All CLL patients had lymphocytosis ranging from 5 × 10^9^/L to 20 × 10^9^/L and had not been treated for at least 3 months prior to blood sampling. The clinical characteristics of our CLL patients are summarized in Table 1. The 22 age-matched healthy individuals included in this study were free of acute or chronic disease.

**Table 1:** Clinical characteristics and outcomes of CLL patients

Patient's ID	Sex	Age (years)	Binet's stage	FISH Aberrations	Treatment	Matutes	sCD23 PVT	Apoptosis in vitro
B118R	Male	89	A	del(13q14) biallelic; del(11q22)monoallelic	yes	5	160	Resistant
G151R	Male	56	A	del(13q14)biallelic	yes	5	ND	Resistant
E147R	Male	70	A	del(13q14)biallelic; del(17p13)monoallelic	yes	4	ND	Resistant
B138R	Male	65	A	del(13q14)monoallelic	yes	5	ND	Resistant
RM1	Male	81	A	del(13q14)biallelic; del(17p13)monoallelic	yes	5	105	Resistant
RM2	Male	77	A	del(13q14)biallelic	yes	5	400	Resistant
RM3	Male	56	A	del(13q14)monoallelic	yes	4	102	Resistant
RF1	Female	74	A	del(13q14)biallelic; del(17p13)monoallelic	yes	5	443	Resistant
RF2	Female	67	A	del(13q14)monoallelic; del(17p13)monoallelic	yes	5	72	Resistant
RF3	Female	56	A	del(13q14)biallelic; del(17p13)monoallelic	no	5	ND	Resistant
U231R	Female	76	A	del(13q14)monoallelic	yes	5	ND	Resistant
G244S	Female	63	A	del(13q14)monoallelic; del(17p13)monoallelic	no	5	ND	Sensitive
B229S	Female	78	A	del(13q14)monoallelic	no	5	ND	Sensitive
S240S	Female	67	A	no	no	4	ND	Sensitive
SF1	Female	80	A	ND	no	4	70	Sensitive
SF2	Female	62	A	ND	no	4	28	Sensitive
SF3	Female	84	A	ND	no	5	97	Sensitive
SM1	Male	82	A	ND	no	4	52	Sensitive
SM2	Male	65	A	no	no	5	high	Sensitive
SM3	Male	74	A	del(13q14)monoallelic	no	4	low	Sensitive
L130S	Male	77	A	del(13q14)monoallelic	no	4	ND	Sensitive
S152S	Male	77	A	ND	no	4	458	Sensitive
B130S	Male	77	A	del(13q14)monoallelic	yes	4	93	Sensitive
B118S	Male	89	A	del(11q22) monoallelic	yes	4	160	Sensitive
M124S	Male	83	A	ND	no	5	ND	Sensitive

Apoptosis score was established 24 h after cell exposure to 10 Gy of *γ*-rays. Patients were considered sensitive (S) when the number of apoptotic cells was at least twofold higher in irradiated cells than in non-irradiated at 24 h of cell culture, or as resistant (R) when no difference was observed between irradiated and non-irradiated cells. Patients RM2 and B118S/R shifted from S to R status. Matutes scoring system was established to distinguish CLL from other lymphoproliferative disorders. This score is based on the immunophenotypic analysis of five markers: CD5^+^, CD23^+^, FMC7^- ^and CD79b^-^, weak expression of monotypic κ or *λ *light chain. A value of 1 is assigned to a given marker with a level typical of CLL, and a total Matutes score at 4 or 5 indicates diagnosis of typical CLL. ND, not determined. Chromosomal aberrations were established by FISH using LSI ATM/LSI p53 and LSI D13S319/LSI 13q34/CEP12 probe sets (Abbott, France) which are complementary to 11q22.3, 17p13.1, 13q14.3, 13q34 and 12p11.1-q11 genome regions, respectively. To be taken into account, the aberration should be present in at least 5% of nuclei.

This study was approved by the Pitié-Salpêtrière Ethics Committee and the CEA Review Board.

### RNA extraction, DNA arrays and analysis

Total RNA was extracted using RNA-Now (Biogentex, Seabrok, TX) according to the supplier's instructions. RNA quality was checked using a Bioanalyzer 2100 (Agilent technologies, Santa Clara, CA). Two series of analyses were performed that included male and female cell samples. Each series comprised RNAs obtained from three resistant and three sensitive CLL samples, and three healthy donors. Three pools were prepared by mixing equal quantities of each set of samples. Gene expression profiling used the Human U133 Set A-B GeneChip for CLL male samples, and the Human U133 2.0 Plus GeneChip (Affymetrix, Santa Cruz, CA) for all other specimens. GeneChip array design and detailed protocols for microarray hybridization are available from Affymetrix. The fluorescence intensity was measured for each microarray and results were normalized via the robust GCRMA procedure (multiple-array average with adjustment for GC content of probes and quantile normalization) using R software to compare the different GeneChips [18]. According to standardized guidelines [19] significantly up- or down-regulated genes were selected using a SAM analysis module [20] with a median false-discovery rate of 1% and a 2-fold change. To avoid any bias in comparison of gene expression between males and females, only genes represented on the U133 set A-B array have been kept after GCRMA procedure and before SAM analysis.

### Validation of microarray data by real-time quantitative RT-QPCR

The expression of 10 randomly selected genes significantly related to CLL, apoptosis resistance and/or gender status was analyzed in additional samples from 12 CLL patients and 6 healthy donors. The genes included *CCL3*, *CD86*, *CNTNAP2*, *CTBP1*, *LSM3*, *MVK*, *RELB*, *SC4MOL*, *TMEM33 *and *UBE2D1 *and were analyzed by real-time quantitative RT-QPCR using an ABI PRISM 7000 Sequence Detection System with Taqman probes and the manufacturer' conditions (Applied Biosystems). Briefly, 1 μg of total RNA was incubated at 70°C for 3 min in the presence of anchored oligo dT primers (ABgene, Epsom, UK), and then reverse transcribed at 42°C for 1 hour in the presence of 250 U M-MuLV reverse transcriptase (ABgene). Quantitative RT-QPCR was performed with Taqman technology using 5 μl of diluted cDNA (1:50), 1.25 μl of the gene expression array and 12.5 μl of Absolute QPCR ROX mix (ABgene) in a final volume of 25 μl. Amplification included an initial denaturation at 95°C for 10 min, followed by 45 cycles at 95°C for 15 s and 60°C for 1 min. Results were normalized to the β-actin gene (ACT) which was simultaneously amplified.

### *In situ *immunofluorescent heterochromatin labeling

The B-cells used for immunofluorescent labeling of HP1α, 3metH3K9 and 5metC were prepared as previously described [21]. Cells were incubated with HP1α, 3metH3K9 (Upstate Biotechnology, Lake Placid, NY) or 5metC (Abcam, Cambridge, UK) (1:100) antibodies overnight at 4°C, followed by incubation for 1 h with a Cy3-conjugated goat anti-rabbit secondary antibody (Jackson ImmunoResearch, West Grove, PA) to visualize 3metH3K9, and FITC-conjugated rabbit anti-mouse IgG (Jackson ImmunoResearch, West Grove, PA) to visualize HP1α-associated chromatin or DNA methylation. After staining with DAPI, slides were mounted with p-phenylenediamine (Sigma Aldrich, Steinheim, Germany). Quantification of 3metH3K9 and HP1α foci/nucleus and measurements of 5metC areas were performed on projections of a Z-stack of 40 images using a 100X objective of an Olympus IX81 microscope and a Coolsnap HQ camera (Princeton Instruments). Image analysis used MetaMorph software (Ver 6.32; Molecular Devices). Quantification of H3K9, HP1 or 5metC labeling was performed for 3D objects in a stack with a minimum voxel number of 15. The differences of total labeling between samples (obtained by summing the volumes of 3D objects) reflected the differences in the distribution patterns or labeling levels. Differences in mean areas were considered significant for a p-value < 0.01 by the Student *t*-test.

### Immunoblotting analysis

Total proteins were extracted from 5 × 10^6 ^B cells from RM (n = 10), SM (n = 7), RF (n = 6) and SF (n = 9) subsets as described previously [22]. Proteins (10 μg) were separated by SDS-PAGE and transferred onto an Immobilon transfer membrane (Millipore-France). Membranes were incubated in TBS with 5% non-fat milk containing antibodies against RelB (Cell Signaling Technology, Danvers, MA) or actin (Thermo Scientific, France). Following staining with secondary antibodies, the proteins were detected with Supersignal West Pico chemiluminescent substrate (Pierce, Rockford, IL). Image Quant software (Amersham France) was used to quantify signal intensities and RelB levels were normalized with β-actin.

### Bisulfite genomic sequencing

Genomic DNA (500 ng) from CLL samples and normal controls was converted with bisulfite using the EZ DNA methylation kit (Zymo Research Corp, CA) according to the manufacturer's instructions. The sequence extending from the -259 bp region of the promoter to the +169 bp of exon 1 was amplified using 2 μl of bisulfite DNA, 2.5 units of AmpliTaq Gold polymerase (Applied Biosystems), and *RELB *forward (5'-GTGATGGTTTTAAGTAGG-3') and reverse (5'-CCAAAACTAACCCAAAC) primers. Amplification conditions included an initial denaturation at 95°C for 10 min, followed by 30 cycles at 95°C for 30 s, 55°C for 30 s, and 72°C for 45 s. For bisulfite sequencing, the PCR products were purified from a 1% agarose gel using the Qiagen Gel Extraction kit (Qiagen France) and subcloned using the p-GEMT Easy Vector system (Promega, Madison, WI). Five clones per sample (two patients per each CLL subset) were then sequenced.

### Chromatin immunoprecipitation (ChIP) and Q-PCR of anti-3metH3K9 and anti-acetyl-H3 immunopurified chromatin

**Figure 1:**
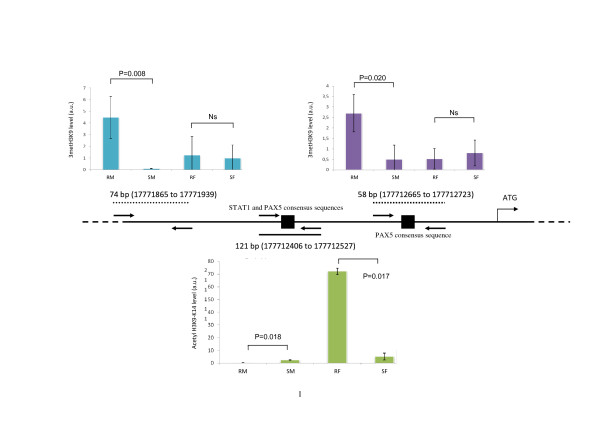
**Epigenetic modifications of histone H3 and RelB expression: histone H3K9 trimethylation in the *RELB *promoter (using ChIP and Q-PCR)**. Chromatin was immunopurified by anti-3meH3K9 immunoprecipitation (ChIP) and the levels of 3metH3K9 in the three promoter regions of *RELB *were established by Q-PCR SYBR Green. These levels were expressed as the percentage of the input chromatin fraction defined in Material and Method section. Ns: non significant. The sequences of the primers used for amplifying the three indicated *RELB *promoter regions are: 74 bp amplicon: forward primer 5' CTGGTGATAGGGATGAGC 3' and reverse primer 5' CGAATGGCAGCAGTGTA 3'; 121 bp amplicon: forward primer 5' GGGTTACAACAACGCACAA 3' and reverse primer 5' CCTCCAAGGTCTCGCTAC 3'; 58 bp amplicon: forward primer 5' TGCTCAATGGGTAAGGC 3' and reverse primer 5' TGCTCTGGACGAGACAAC3'.

**Figure 2:**
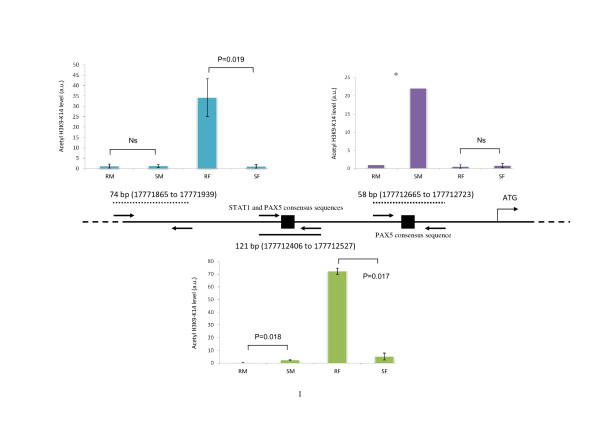
**Epigenetic modifications of histone H3 and RelB expression: histone H3K9-K14 acetylation in the *RELB *promoter (using ChIP and Q-PCR)**. Chromatin was immunopurified by anti-3meH3K9 or anti-acetyl-H3 immunoprecipitations (ChIP) and the levels of 3metH3K9 and acetyl-H3 in the three promoter regions of *RELB *were established by Q-PCR SYBR Green. These levels were expressed as the percentage of the input chromatin fraction defined in Method section. Ns: non significant; *for resistant male samples the values for the level of acetyl-H3 were too low to allow the calculation of the percentages. The sequences of the primers used for amplifying the three indicated *RELB *promoter regions were the same as described for histone H3K9 trimethylation (Figure 1).

Preliminary assays allowed us to determine the immunoprecipitation conditions to ensure that the amount of antibody and percentage of cross-linking agent were not limiting in the ChIP yields. Freshly isolated B lymphocytes (4 × 10^7^) from RM (n = 6), SM (n = 6), RF (n = 6) and SF (n = 6) were cross-linked for 10 min at 37°C in 0.5% formaldehyde. Fixed cells were washed twice with ice-cold PBS and lysed in 400 μl of ChIP lysis buffer for 10 min (Tris 50 mM pH 8, EDTA 10 mM, SDS 1%). Chromatin fragments between 250 bp and 3000 bp were then produced by sonication with a Branson sonifier 450. Five μg of 3metH3K9 or acH3K9/acH3K14 antibodies (#07-442 and #06-599, respectively, Upstate Biotechnology, CA, USA), were used to precipitate 1.5 μg of fragmented chromatin measured using Quant it PicoGreen dsDNA Reagent (Molecular Probes, Invitrogen, France). Immunoprecipitations were carried out in buffer containing 0,1% SDS, 1% Triton X-100, 2 mM EDTA, 20 mM Tris-HCl (pH 8,0), 150 mM NaCl and a protease inhibitor cocktail at 4°C overnight. A negative control without antibody was run simultaneously with the samples. Immunocomplexes were collected by adsorption to protein A sepharose CL-4B (Amersham) beads blocked in 0.5% BSA according to the manufacturer's protocol. Washing and elution of immunocomplexes were carried out as described elsewhere [23]. Cross-linking was reversed for 5 h at 65°C and samples were then treated with proteinase K. Immunoprecipitated DNA was extracted with phenol-chloroform-isoamyl alcohol. Three regions of the *RELB *promoter were assessed for the presence of trimethylated histone H3 using Q-PCR. The location of promoter *RELB *and primer sequences are indicated in Figure 1 and Figure 2. Amplification was performed using 12.5 μl of absolute QPCR SYBR Green ROX mix (Thermo Scientific France), 10 μM of each primer and 100 ng of DNA in a final volume of 25 μl. Amplification conditions included an initial denaturation at 95°C for 15 min, followed by 30 cycles at 95°C for 15 s, 56.2°C for 30 s, and 72°C for 30 s. Serial dilutions of control DNA ranging from 200 to 0.02 ng were used for quantification of the signal. The levels of 3metH3K9 and acetyl-H3 in the three promoter regions of *RELB *were expressed as the percentage of the input chromatin fraction defined by the following equation: (immunoprecipitated chromatin fraction with antibody-chromatin fraction without antibody)/input chromatin fraction.

## Results

### The gene expression signatures for apoptosis resistance in CLL differ between males and females

Comparisons of gene expression profiles between resistant and sensitive CLL subsets were assessed separately for males and females. In males, the vast majority of the genes had lower expression levels in resistant compared with sensitive CLL samples (463 out of 472 genes, 98%) (Additional file 1, Figure S1a). In contrast, over- and under-expressed genes in females were equally represented in resistant (475 out of 803 genes, 59%) and sensitive samples (328 out of 803 genes, 41%) (Additional file 1, Figure S1b). Only 17 genes that distinguished resistant from sensitive CLL were found to be common between male and female patients. Remarkably, for three of these genes (*RELB*, *CTBP1*, and *TMEM33*), the relative expression levels reflected by the R vs. S ratios were opposite between male and female patients (Table 2). Moreover, as evidenced from the comparative analysis of transcriptome profiles from healthy male and female control samples (Additional file 1, Figure S1c), the set of 419 significant differentially expressed genes included only one of the above 17 genes (CACNA1A). Additional file 1 Table S1 shows that a majority of NFκB target genes appeared as down-regulated in resistant males' while an upregulation was observed in females' samples. Taken together, these data suggest that the gene expression signature for resistance is dependent upon the sex of the patient. To validate these microarray data, we further performed RT-QPCR for 10 randomly selected genes in an additional 12 CLL patients and 6 healthy donors (Additional file 1, Figure S2) including the *RELB*, *TMEM33 *and *CTBP1 *genes.

**Table 2:** Significantly up- and down-regulated genes that discriminate resistant (R) from sensitive (S) CLL patients and that are common to males (M) and females (F)

Gene name*	Gene Symbol	Chromosome Location	Fold change RM vs. SM	Fold change RF vs. SF
trafficking protein, kinesin binding 2	TRAK2	2q33	0.054	0.379
calcium channel, voltage-dependent, P/Q type, alpha 1A subunit	CACNA1A	19p13.2-p13.1	0.116	0.135
contactin associated protein-like 2	CNTNAP2	7q35-q36	0.003	0.060
**C-terminal binding protein 1**	**CTBP1**	**4p16**	**0.290**	**2.344**
discs, large (Drosophila) homolog-associated protein 4	DLGAP4	20q11.23	0.164	0.389
DNA polymerase-transactivated protein 6	DNAPTP6	2q33.1	0.199	0.177
developmentally regulated GTP binding protein 2	DRG2	17p11.2	0.320	0.442
G protein pathway suppressor 2	GPS2	17p13	0.430	0.425
HOM-TES-103 tumor antigen-like	HOM-TES-103	12p13.3	0.303	0.446
integrin, alpha E (antigen CD103, human mucosal lymphocyte antigen 1; alpha polypeptide)	ITGAE	17p13	0.450	0.434
phosphoribosylformylglycinamidine synthase (FGAR amidotransferase)	PFAS	17p13.1	0.320	0.457
pleckstrin homology domain containing, family B (evectins) member 1	PLEKHB1	11q13.5-q14.1	0.203	0.484
RNA binding motif, single stranded interacting protein 1	RBMS1	2q24.2	0.036	0.352
**v-rel reticuloendotheliosis viral oncogene homolog B, nuclear factor of kappa light polypeptide gene enhancer in B-cells 3**	**RELB**	**19q13.31-q13.32**	**0.245**	**3.033**
**transmembrane protein 33**	**TMEM33**	**4p13**	**0.203**	**2.013**
vesicle-associated membrane protein1, synaptobrevin1	VAMP1	12p	0.287	0.395
vanin 2	VNN2	6q23-q24	0.434	0.424

*Genes listed were identified as being differentially expressed after direct comparisons of gene expression profiles between resistant and sensitive CLL samples for males and females. FC, fold change

### The chromatin organization discriminates CLL patients from healthy individuals, and also resistant from sensitive CLL cell types

**Figure 3:**
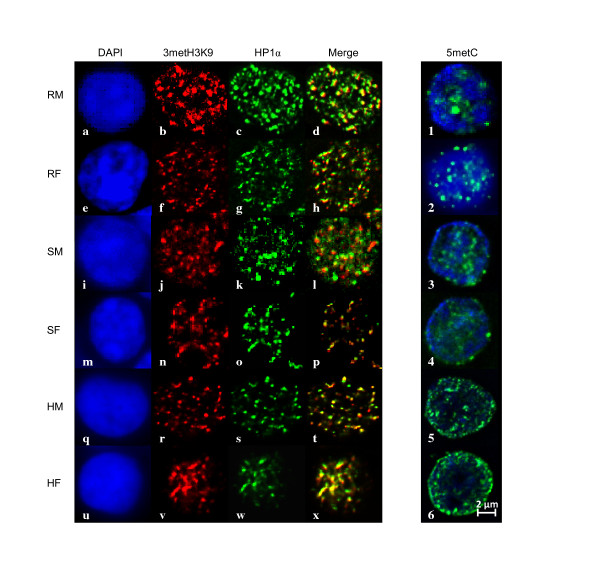
**Distribution of HP1α, 3metH3K9 proteins and 5metC in human CLL cells**. Representative images are shown for the localization of HP1α (a-x). All cells were stained with DAPI (blue) (a,e,i,m,q,u). Cells were stained with a polyclonal antibody against 3metH3K9 (red) (b,f,j,n,r,v) a marker of inactive chromatin and antibody against HP1α (green) (c,g,k,o,s,w) a marker of heterochromatin. Merged images show the co-localization of these two proteins. Labeling with a 5metC antibody (green) (1-6) revealed a global hypomethylation state and a more diffuse pattern in CLL cells (1-4) vs. control cells (5-6). Scale bar, 2 μm.

We addressed the question of whether epigenetic markers for chromatin organization may underlie the observed differences in gene expression according to the resistance or sensitivity status and sex of the patient. A preferential perinuclear localization of 5metC labeling was observed in cells from healthy donors compared with a more diffuse intra-nuclear pattern in CLL cells, irrespective of patient status or sex (Figure 3). This difference may be of particular interest since perinuclear region contains actively transcribed genes whose expression might be controlled by 5metC in normal B cells but much less in CLL cells. The level of 5metC labeling was significantly lower in CLL samples than in healthy tissues (n = 81 *vs. *n = 33 spots; p < 0.001), consistent with the previously described hypomethylation of CLL cells [12]. In addition, resistant individuals showed a higher level of DNA methylation than sensitive cases (n = 42 vs. n = 37 spots; p < 0.01) with stronger 5metC labeling in males than in females (n = 23 vs. n = 19 spots; p < 0.015). This was not due to differences between healthy males and females (n = 18 vs. n = 15 spots; p = 0.07). No differences in the distributions were observed by sex in sensitive individuals.

**Figure 4:**
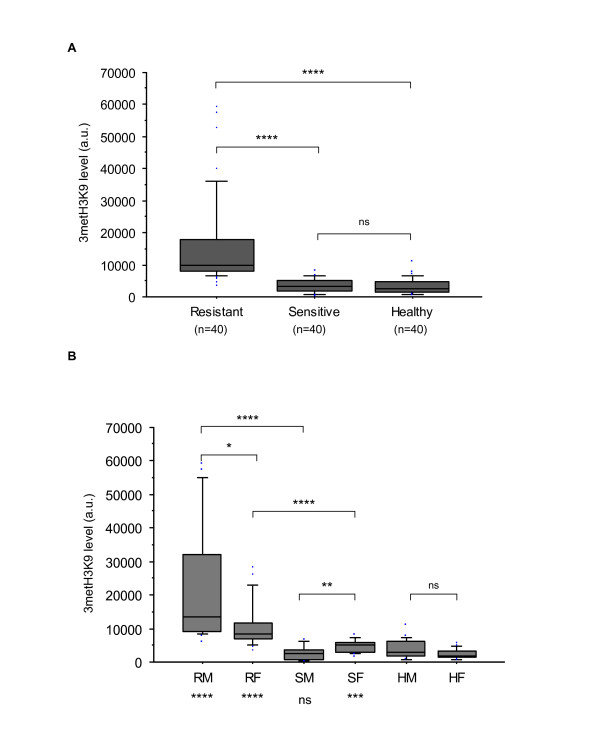
**Comparison of 3metH3K9 levels between resistant and sensitive CLL and healthy cell samples (A) and between each subgroup according to the gender (B)**. For each type of labeling 40 cells for each subgroup were analyzed. 3metH3K9 was at significantly greater level in CLL than in healthy controls, and up-regulated in resistant compared with sensitive CLL patients (arbitrary units). Differences in the mean areas were considered significant for a p-value < 0.01 by Student's *t*-test (A) or U-test (B). Comparisons between each CLL phenotype (n = 20) and their control counterparts are given at the bottom of the figure (i.e. RM vs. HM; RF vs. HF; SM vs. HM and SF vs. HF). Results are presented using box-plots with medians (*lines inside boxes*), 25th and 75th percentiles (*limits of boxes*), and the 10th and 90th percentiles (*whiskers*). Outliers are also indicated. RM, resistant male; RF, resistant female; SH, sensitive male; SF, sensitive female; HM, healthy male; HF, healthy female. ****, p < 0.0001; ***, p < 0.001 < p; **, p < 0.01; *, p < 0.05; ns, not significant.

**Figure 5:**
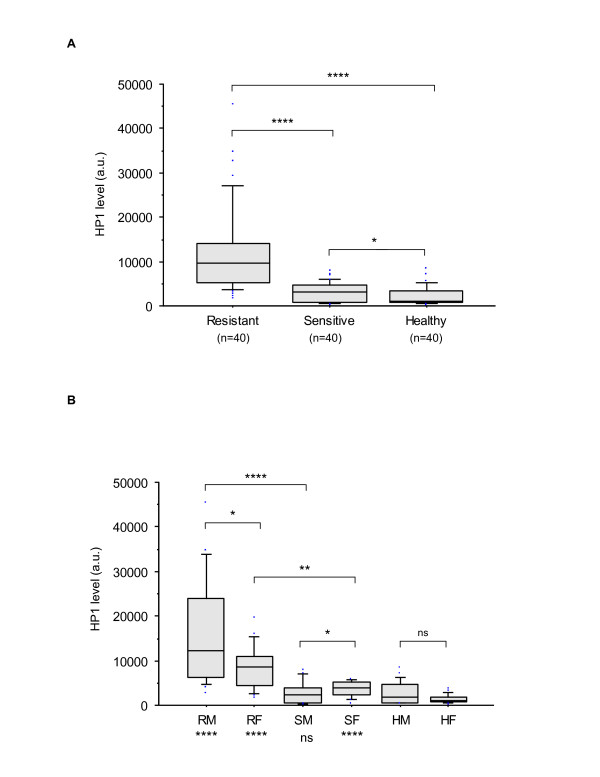
**Comparison of HP1α levels between resistant and sensitive CLL and healthy cell samples (A) and between each subgroup according to the gender (B)**. For each type of labeling 40 cells for each subgroup were analyzed. HP1α was at significantly greater level in CLL than in healthy controls, and up-regulated in resistant compared with sensitive CLL patients (arbitrary units). Statistical analyses and results presentation were the same as described for Figure 2.

Immunofluorescence labeling with HP1α and 3metH3K9, which are known to be associated with heterochromatin formation and the transcriptional repressive state, was also assessed to evaluate structural and functional chromatin organization. Localization of the HP1α isoform within the nucleus of interphase B-cells revealed a different chromatin organization between CLL and healthy samples (Figure 3). The chromatin appeared to be more condensed in resistant cells compared with sensitive cells or control cells (Figure 3). Although the 3metH3K9 or HP1α distributions were similar between healthy male and female cells, they clearly discriminated sensitive from resistant subsets according to sex (Figure 4 and Figure 5, respectively). For all but the sensitive male subset, the distributions of HP1α and 3metH3K9 in CLL differed from the healthy counterparts. These data provide evidence of striking differences in the chromatin organization between CLL subsets with a notably more condensed chromatin in resistant CLL in males.

### Increased 3methyl-H3K9 in the promoter region of the *RELB *gene occurs in resistant male but not resistant female CLL and inversely, increased level of acetyl-H3 occurs in resistant female but not resistant male CLL

**Figure 6:**
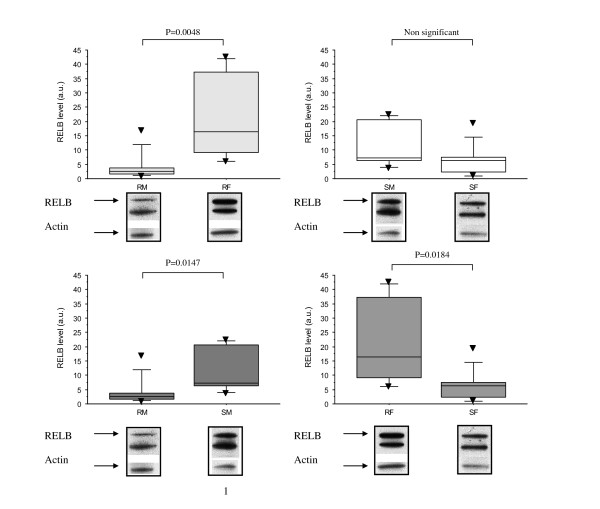
**Western blot analysis of RelB in CLL patients**. The expression of RelB is higher in resistant CLLs in females (RF) (n = 6, 21.2 ± 15.5) than in resistant CLL in males (RM) (4.2 ± 4.8, n = 10), U test p = 0.0048; higher also in resistant females (RF) (n = 6, 21.2 ± 15.5) vs. sensitive females (SF) (6.2 ± 5.5, n = 7), U test p = 0.0184; higher in sensitive males (SM) (12.2 ± 8.0, n = 7) compared with resistant males (RM) (4.2 ± 4.8, n = 10), U test p = 0.0147; but no different between sensitive males (SM, n = 7) and sensitive females (SF, n = 9). Results are shown using box-plots with medians (*lines inside boxes*), 25th and 75th percentiles (*limits of boxes*), and the 10th and 90th percentiles (*whiskers*). (▼) indicates an outlier. RELB values are given as arbitrary units after normalization with β-actin. Representative images of Western blots are shown under the graphs.

Given the differences in the chromatin organization between CLL subsets, we next focused on searching for epigenetic changes at the specific *RELB *locus as this gene was identified as under-expressed in resistant *vs. *sensitive cells in males and over-expressed in resistant cells in females. Consistent with the transcript levels, we found that the levels of the RelB protein were significantly decreased in resistant male CLL cells when compared with sensitive cells. In contrast, the RelB levels were increased in all female CLL subsets (Figure 6). The DNA methylation status of 58 CpG sites located within the *RELB *promoter and exon 1 of this gene was examined using bisulfite genomic sequencing. DNA from healthy donors and CLL patient B cells were treated with sodium bisulfite allowing conversion of unmethylated cytosine to uracil and then used for PCR amplification of a 400 bp sequence. Following cloning of the PCR products, five clones per DNA sample were sequenced.

**Figure 7:**
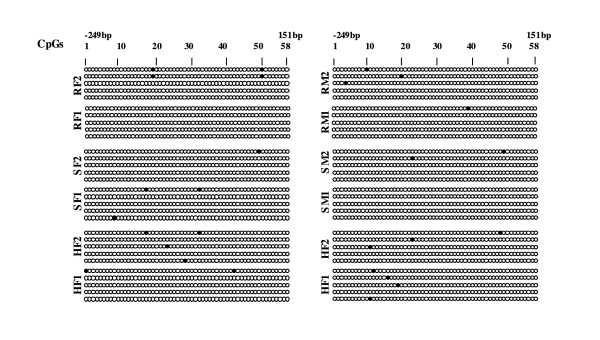
**DNA methylation analysis of the RELB promoter region**. The sequence shown extends from the -259 bp region of the promoter to the +169 bp position in exon 1. R, resistant; S, sensitive; M, male; F, female. White spot, no methylation; black spot, methylation.

As shown in Figure 7, no differences in the DNA methylation of *RELB *between the different CLL subsets were detectable, at least for this 5' region and the first exon. The 3metH3K9 histone modifications that are known to be involved in gene silencing were analyzed by chromatin immunoprecipitation. The total DNAs that cross-linked to 3metH3K9 were then used in RT-QPCR reactions with oligonucleotide primers specific for three regions of the *RELB *promoter (Figure 1) covering a 1 kb 5' upstream region from the transcriptional initiation site. Two of these promoter regions contain the consensus DNA binding site for the STAT3 or PAX5 transcription factors (Figure 1 and additional file, Table S2). The results showed a significant increase in the 3metH3K9 levels in resistant male but not in female CLL samples (Figure 1). In parallel, the level of acetyl-H3 (antibody recognizes acetylated lysine K9 and K14), used as a positive epigenetic marker associated with increased gene expression, showed low level in male and high level in resistant female samples. Moreover, in female CLL, the increase of acetyl-H3 level in two RELB promoter regions (74 bp and 121 bp) is significant when resistant samples were compared to sensitive (Figure 2).

**Figure 8:**
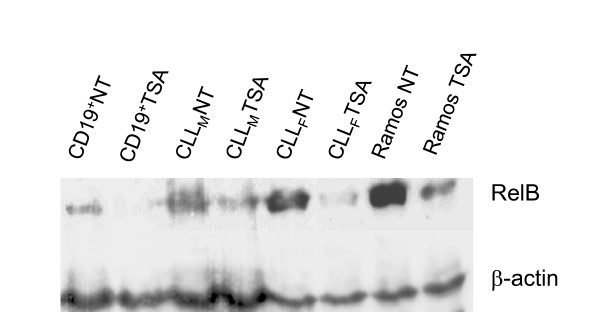
**Down-regulation of RelB expression by HDAC inhibitor TSA**. Primary B cells from healthy donor (CD19^+^), cells from male or female CLL patients (CLL_M _and CLL_F_, respectively), or Ramos B cell line (established from Burkitt lymphoma's patient), used as control, were untreated (NT) or preincubated for 16 hr with 200 nM trichostatin A (TSA). Total protein fraction was prepared as described in Methods section. Western blot analyses show that TSA induced RelB down-regulation in all B cell types tested.

To further explore if *RELB *expression may be under an epigenetic control we have sought whether it may be modified by HDAC inhibition. For this purpose we have pretreated primary CD19^+ ^B cells from healthy donor, CLL cells and Ramos B cell line with a general HDAC inhibitor trichostatin A (TSA) at 200 nM for 16 hrs and then performed Western blot analysis of total cellular level of RelB. As shown (Figure 8), HDAC inhibition resulted in a decreased level of RelB in all B cell types tested.

## Discussion

The most relevant contribution to date of microarray technology to the biology of CLL is certainly the identification of a homogeneous phenotype related to memory B cells [9] and its classification as a distinct type of B lymphoma [24]. In particular, for a given CLL subset defined by disease aggressiveness, this method may provide new insights into mechanisms that have yet to be revealed. In addition to transcriptional changes defined by the microarray approach, it has become evident that epigenetic alterations that determine structural and functional chromatin organization should be integrated into these types of studies. Indeed, molecular profiling in CLL has allowed the identification of new genes for which the expression is dependent on CpG island methylation [13]. The down-regulation of the death-associated protein kinase 1 (DAPK1) gene in CLL indicates that both genetic and epigenetic factors may define both the sporadic and inherited forms of this disease [17].

In our current study, we employed a microarray methodology to search for potential candidate genes that may discriminate aggressive cases of CLL according to the sex of the patient. In our previous studies, we reported that in aggressive forms of CLL, the cells display partially defined mechanisms enabling them to avoid apoptotic death following DNA damage [7,22,25]. Thus, comparing resistant and sensitive CLL samples according to patient sex, the gene expression profile of resistant cells in male patients was found unexpectedly to differ from females. This suggests that analyses based on cellular susceptibility to DNA damage-induced apoptosis combined with patient sex may reveal a new sub-class of CLL that has previously been unknown.

CLL cells that are resistant to DNA damage-induced apoptosis may harbor mutated as well as wild type IgV_H _genes, mutated or wild-type TP53, and any different (probably multiple) types of chromosomal aberrations. In contrast, sensitive cells contain wild-type TP53 only and any (but probably a unique) type of chromosomal aberration [25] (Table 1). These biological markers have previously been used as criteria to select and to class CLL samples for microarray analyses but are not necessarily linked to the susceptibility of CLL cells to DNA damage-induced apoptosis. We thus evaluated whether this last type of CLL classification may be useful in identifying new molecular markers which have not been elucidated using other characteristics of this disease.

Remarkably, when male and female CLL samples were compared in terms of their ability to undergo apoptosis, all but 2% of the genes in the male samples were under-expressed in resistant compared with sensitive CLL types. In contrast, 41% of the genes were under-expressed in resistant female CLL samples (Additional file 1, Figure S1). The significant difference between resistant male and female CLL is emphasized by the very low number of genes in common, (17 in total) between these subsets (Table 2). Thus, it was striking that in the resistant male subset, 98% of the genes were down-regulated. Based on this observation, we addressed whether global chromatin condensation could underlie this transcriptional repression. We used immunofluorescence *in situ *labeling of a repressive chromatin state to evaluate the global levels of DNA and histone methylation in the sample CLLs (Figure 3). Immunofluorescence staining with HP1α, 3metH3K9 and 5metC showed differences in the distribution of inactive chromatin markers between resistant and sensitive cells and is indicative of a global repressive state of chromatin in the resistant male CLL subset. Indeed, HP1α, 3metH3K9 and 5metC are associated with heterochromatin-driven transcriptional repression [16].

These observations are in agreement with recently described features of CLL that involve altered epigenetic control i.e. the over-expression of nucleolin and its cytoplasmic retention in CLL cells [26] and an altered telomere length in a subset of CLL with a poor prognosis [27]. Indeed, the nucleolar structure involving nucleolin and telomere lengths has been shown to be controlled by epigenetic factors, such as H3K9 methylation [28,29]. *RELB *was elucidated as one of three discriminatory genes since its relative expression, reflected by the ratio R/S, was opposite between male and female CLL. The level of histone H3K9 trimethylation significantly increased in three promoter regions of *RELB*, two of them contain potential DNA-binding sites for the STAT3 and PAX5 (Figure 1, see also additional file Table S2) transcriptional factors that may be blocked by this type of histone modification. Of note, this region contains the motifs for 20 TFs which may be potentially involved in the *RELB *transcriptional control. The observation that the level of acetyl-H3, used as the marker of positive gene expression, was extremely low in male samples while in resistant female samples it was significantly high (Figure 2), further highlighted the role of epigenetic differences between male and female CLL samples. Hence, in the same promoter regions, histone modifications were not found to be associated with DNA methylation (Figure 7). Effectively, DNA methylation was investigated in regions that were more proximal from TSS and first exon than those investigated for 3metH3K9. According to UCSC Genome Browser http://genome.ucsc.edu on Human Mar. 2006 (NCBI36/hg18), the assembly used for this study, *RELB *gene position was 50,196,552 - 50,233,292 and mRNA at 50,196,535. The most proximal CpG islet is located 50,196,420 - 50,197,139. This means that the most probable site for DNA methylation is located -115 bp upstream TSS. The region of the *RELB *locus that was bisulfite sequenced was a 428-bp region comprising the promoter (from -259 bp) and first exon (to +169 bp). Thus, we probably cover the region that is the most likely affected by DNA methylation.

DNA methylation could be correlated (but not necessarily) with histone H3K9 modifications and vice versa. This discrepancy may be due to the fact that histone methylation is not always overlapping with CpG islands regions. The fact that we investigate upstream sequences for histone modifications might lead to under-estimate a putative 3metH3K9 modification in the sequences investigated for DNA methylation, and this results that *RELB *could be even more silenced than we proposed. Therefore, our findings support that H3K9 methylation rather than deoxycytosine methylation is responsible for *RELB *silencing.

As learned from the expression regulation of some autosomal genes, genes that are specifically maternally repressed may be CpG-hypomethylated to the same extent as paternally over-expressed genes. This suggests that DNA methylation is not necessarily modified in the transcriptionally "open" nucleosomal state at the promoter region [30]. In addition, an impairment of DNA and histone modifications has been observed in a mouse leukemia cell line, L1210, where an inverse relationship in the regulation of the silencing of candidate genes was reported [31]. This suggests that the methylation of DNA and histones is less cooperative in hematological cancers compared with other types of malignant cells. We recently reported that an epigenetic heterochromatinisation may be the mechanism involved in telomere shortening in both female- and male-derived resistant CLL cells [32,33] resulting in an aberrant chromatin structure of telomeric regions. Together with current data, it appears evident that there is an altered epigenetic control involved in heterochromatinisation in CLL cells. While B cell lineage differentiation have been shown to depend on inherited epigenetic factors it could be speculated that malignant transformation of CLL cells results from an epigenetic defect resulting in a modified expression of *RELB *which is potentially involved in this process. Whether *RELB *inactivation in males occurs concomitantly with malignant B cell transformation, warrants further investigations.

The NF-κB family is a tightly coordinated group of five identified transcription factors with structural homologies enabling them to be involved in two major cellular responses mainly related to stress and inflammation as well as to cancer development and progression [34]. With regards to the aggressiveness of CLL, the RelA member of the NF-κB family, involved in its heterodimeric form with p50 and through its temporal transactivation of "regulon" type genes in a canonical manner, has been recently reported to be associated with both *in vitro *survival and clinical disease progression in CLL. NF-κB pathway activation is achievable exogenously by stress (reactive oxygen species or DNA damage) or by death receptor stimulation. While this activation might results in a pro- or anti-apoptotic signaling we could speculate that the success of therapy would depend on its activity. In consequence, this finding suggests that *RELB *is a promising new therapeutic target for this disease [35]. Moreover, as an NF-κB family member associated with the control of the adaptive immune response and the response to metabolic stress, RelB may be of particular interest in the emergence of progressive form of CLL since the alternative NF-κB pathway is potentially involved in both early and late differentiation of B cells [36,37]. Together with our demonstrations of local heterochromatinization of telomeric regions [32,33] and in agreement with the recently discovered role for the multifunctional transcriptional positive coactivator 4 (PC4) in targeting heterochromatinization in non-neuronal human cells [38], different levels of RelB expression between female and male CLL cells may involve a cell differentiation- and/or gender-specific factor that controls a dynamic state of chromatin region containing *RELB*.

## Conclusion

We have used a microarray approach to analyze the gene expression profiles of a restricted number of CLL samples segregated according to the patient gender and to the cell sensitivity to undergo or not DNA damage-induced apoptosis *in vitro*. This experimental plan allowed us to identify *RELB *as the new gene epigenetically down-regulated in a sex-dependent manner in cells resistant to apoptosis. This was subsequently confirmed and validated in a larger cohort of CLL samples. Whereas the incidence of CLL is also sex-dependent, the origin of CLL cell remains still unknown and the disease remains still incurable, these findings should initiate a development of new approaches targeting RelB and alternative NF-κB pathway that might be discriminative between CLL leukemogenesis in males and females.

## Competing interests

The authors declare that they have no competing interests.

## Authors' contributions

J-B M and JD designed and performed research and wrote the paper; OR performed research and wrote the paper; TB, NG, MK, TFO, FNK and SC contributed new analytical tools and analysed data; LV, FNK and HMB designed research and managed clinical data. All authors reed and approved the manuscript.

## Pre-publication history

The pre-publication history for this paper can be accessed here:

http://www.biomedcentral.com/1755-8794/3/53/prepub
